# The Regulated Cell Death and Potential Interventions in Preterm Infants after Intracerebral Hemorrhage

**DOI:** 10.2174/1570159X21666221117155209

**Published:** 2023-05-18

**Authors:** Yanan Wu, Yanyan Sun, Xiaoyang Wang, Changlian Zhu

**Affiliations:** 1 Henan Key Laboratory of Child Brain Injury and Henan Pediatric Clinical Research Center, Third Affiliated Hospital and Institute of Neuroscience of Zhengzhou University, Zhengzhou 450052, China;; 2 Department of Human Anatomy, School of Basic Medical Science, Zhengzhou University, Zhengzhou, China;; 3 Centre for Perinatal Medicine and Health, Institute of Clinical Sciences, Sahlgrenska Academy, University of Gothenburg, Gothenburg, Sweden;; 4 Center for Brain Repair and Rehabilitation, Institute of Neuroscience and Physiology, Sahlgrenska Academy, University of Gothenburg, Gothenburg, Sweden

**Keywords:** Intracerebral hemorrhage, preterm infant, secondary brain injury, regulated cell death, ferroptosis, PANoptosis

## Abstract

Intracerebral hemorrhage (ICH) in preterm infants is one of the major co-morbidities of preterm birth and is associated with long-term neurodevelopmental deficits. There are currently no widely accepted treatments to prevent ICH or therapies for the neurological sequelae. With studies broadening the scope of cell death, the newly defined concept of regulated cell death has enriched our understanding of the underlying mechanisms of secondary brain injury after ICH and has suggested potential interventions in preterm infants. In this review, we will summarize the current evidence for regulated cell death pathways in preterm infants after ICH, including apoptosis, necroptosis, pyroptosis, ferroptosis, autophagy, and PANoptosis as well as several potential intervention strategies that may protect the immature brain from secondary injury after ICH through regulating regulated cell death.

## 1 INTRODUCTION

Advances in health care have led to increased survival of preterm infants, especially extremely premature infants born before gestational week 28. However, about one-third of preterm infants born before 30 weeks of gestation are affected by intracerebral hemorrhage (ICH) [1, 2]. Germinal matrix-intraventricular hemorrhage is the type of ICH that typically occurs in preterm infants, and the underlying pathogenic mechanisms that lead to this type of hemorrhage are the fragility of the vasculature and fluctuation of the blood flow in the germinal matrix [3]. The germinal matrix is a specialized area where neuronal and glial cell differentiation takes place and is present in the brain between 8 and 36 weeks of gestation in humans. It is highly vascularized in the preterm infant and is essentially absent by term, thus explaining the increased vulnerability to ICH at lower gestational ages [2, 4]. The microvasculature network of the germinal matrix is immature in the basal lamina and lacks adequate autoregulation of the cerebral blood flow, and thus fluctuations in blood flow can result in rupture of blood vessels and subsequent hemorrhage. The hemorrhage may be confined to the germinal matrix region or it may extend into the adjacent ventricular system or extend into the white matter [5]. ICH is an important complication of preterm infants and is the major cause of neonatal morbidity and mortality, and the incidence and severity are inversely associated with gestational age and birth weight [6, 7]. There is currently no widely accepted therapy to prevent ICH or treat its sequelae although erythropoietin has been shown a reduce the likelihood of poor outcomes [5, 7]. Most survivors of ICH develop neurological disabilities and/or psychiatric disorders including cerebral palsy, mental retardation, problems with executive function, and/or hydrocephalus, which negatively affect their quality of life and working capacity [8].

The mechanisms of brain injury in preterm infants after ICH are complex and remain largely unknown. However, it is generally believed that ICH-induced brain injury includes primary injury *via* physical disruption and mass effect and secondary brain injury through the release of hemoglobin/heme and free iron-induced neurotoxicity [9-12]. Neural cell death after ICH occurs during both phases of injury, but the major difference is that the cell death is mainly accidental cell death or necrosis in the primary phase of brain injury, while regulated or programmed cell death is the main form in the secondary brain injury phase, which is associated with neurological outcomes [13]. The primary injury occurs immediately and accidentally after hemorrhage, while secondary brain injury starts hours after injury and lasts several days and even months thus causing more serious and extensive damage. Thus secondary brain injury after ICH has drawn much attention in the study of post-hemorrhagic cell death mechanisms and for developing specific cell death inhibitors to prevent post-ICH brain injury.

The immature brain possesses programmed cell death machinery, and regulated cell death is more prominent after insult. There is currently no systematic review regarding preterm ICH-induced regulated cell death despite several articles that have reviewed the progress of regulated cell death in adult ICH. However, there are distinct differences between mature brains and immature brains, which indicates that the immature brain may be predisposed to some type of regulated cell death after ICH. This review aims to summarize the current progress of ICH-induced regulated cell death and potential therapeutic strategies in the immature brain.

## 2 PATHOPHYSIOLOGY OF ICH IN THE IMMATURE BRAIN

**Fig. (1):**
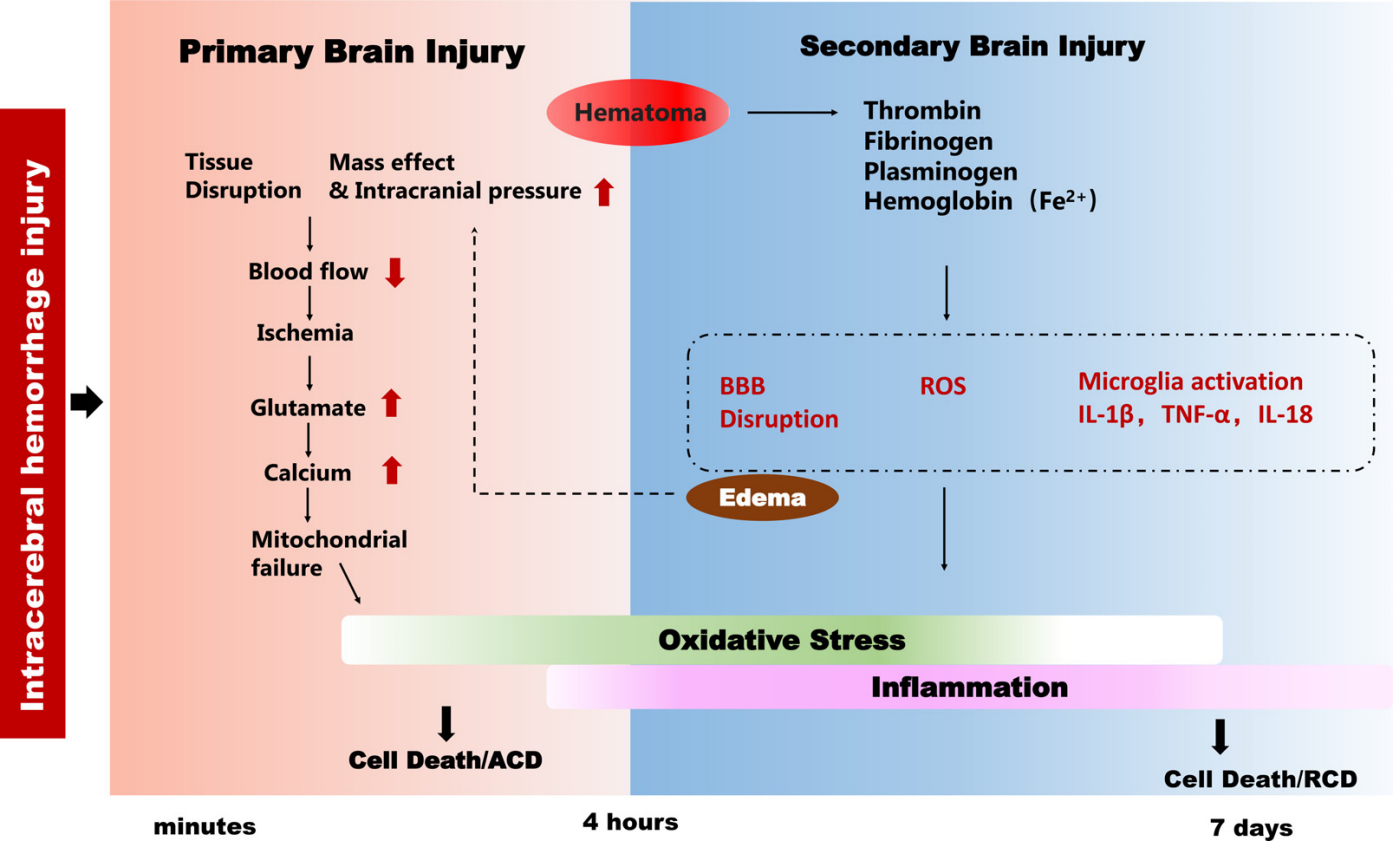
The pathophysiology of ICH brain injury. The pathophysiology of ICH evolves over hours to days and is marked by two phases of injury – primary brain injury and secondary brain injury. Hematoma develops within the first 4 hours after hemorrhage and lasts for several days, and it can cause a mass effect and increase intracranial pressure (ischemia), which then induces a series of cellular stress responses and causes accidental cell death, and this process is referred to as primary brain injury. Secondary brain injury results from the blood components or the products of clot lysis that trigger microglial activation and ROS generation together with BBB disruption and edema formation. These toxic events ultimately lead to brain cell death through regulated cell death.

Primary brain injury follows acute bleeding through physical compression, and the hematoma may cause temporary obstruction of cerebral spinal fluid (CSF) drainage and lead to ventricular dilatation. Clearing the hematoma after ICH can reduce the physical compression and toxic effects of the extravasated blood. Red blood cells lyse and release toxic components from the hematoma including thrombin, platelet, fibrinogen, extracellular hemoglobin, heme, and free iron into the brain [3, 14]. These toxic substrates can trigger microglia activation and immune cell invasion, oxidative stress, and the release of pro-inflammatory molecules, all of which can activate cell death signals (Fig. **1**). Besides traditional regulated cell death pathways like apoptosis, necroptosis, and autophagy, some other regulated cell death pathways have recently been defined, such as pyroptosis, ferroptosis, and PANoptosis, and these different cell death types are precisely regulated through separate signaling pathways, but with extensive crosstalk [15].

The influence of age on brain injury is a prominent feature in the developing brain; for example, intraventricular hemorrhage mainly occurs in preterm infants and is negatively correlated with gestational age [5, 16]. Compared to the adult brain, the immature brain has a high rate of oxygen consumption, a high concentration of unsaturated fatty acids, and a low concentration of antioxidants, and thus it is particularly sensitive to oxidative stress leading to cell death [17, 18]. It has been identified that the immature brain is more vulnerable to the detrimental effects of blood components compared to the adult brain [19]. The influence of age on cell death mechanisms has been reported, and apoptotic cell death is more pronounced in the immature brain than in juvenile and adult brains, while autophagy is more pronounced in the adult brain [20]. Further study showed that the key organelle involved in cell death, the mitochondrial membrane permeability transition pore, plays different roles in the neonatal and adult brain after insult [21]. In addition to cell death, the regenerative responses after insult also show age dependence with stronger regenerative response in the neonatal brain than in the juvenile brain, while microglia activation and inflammation are much stronger in the juvenile brain than in the neonatal brain [22]. Furthermore, neonates, especially preterm infants, have higher ratios of fetal hemoglobin, which could influence peroxidase activity [23]. These developmental differences in pathophysiological changes will induce different types of neural cell death after hemorrhage and thus attempts to develop prevention and treatment strategies to inhibit regulated cell death and brain injury need to match the developmental stage.

## 3 CURRENT EVIDENCE OF REGULATED CELL DEATH IN THE IMMATURE BRAIN AFTER ICH

**Fig. (2):**
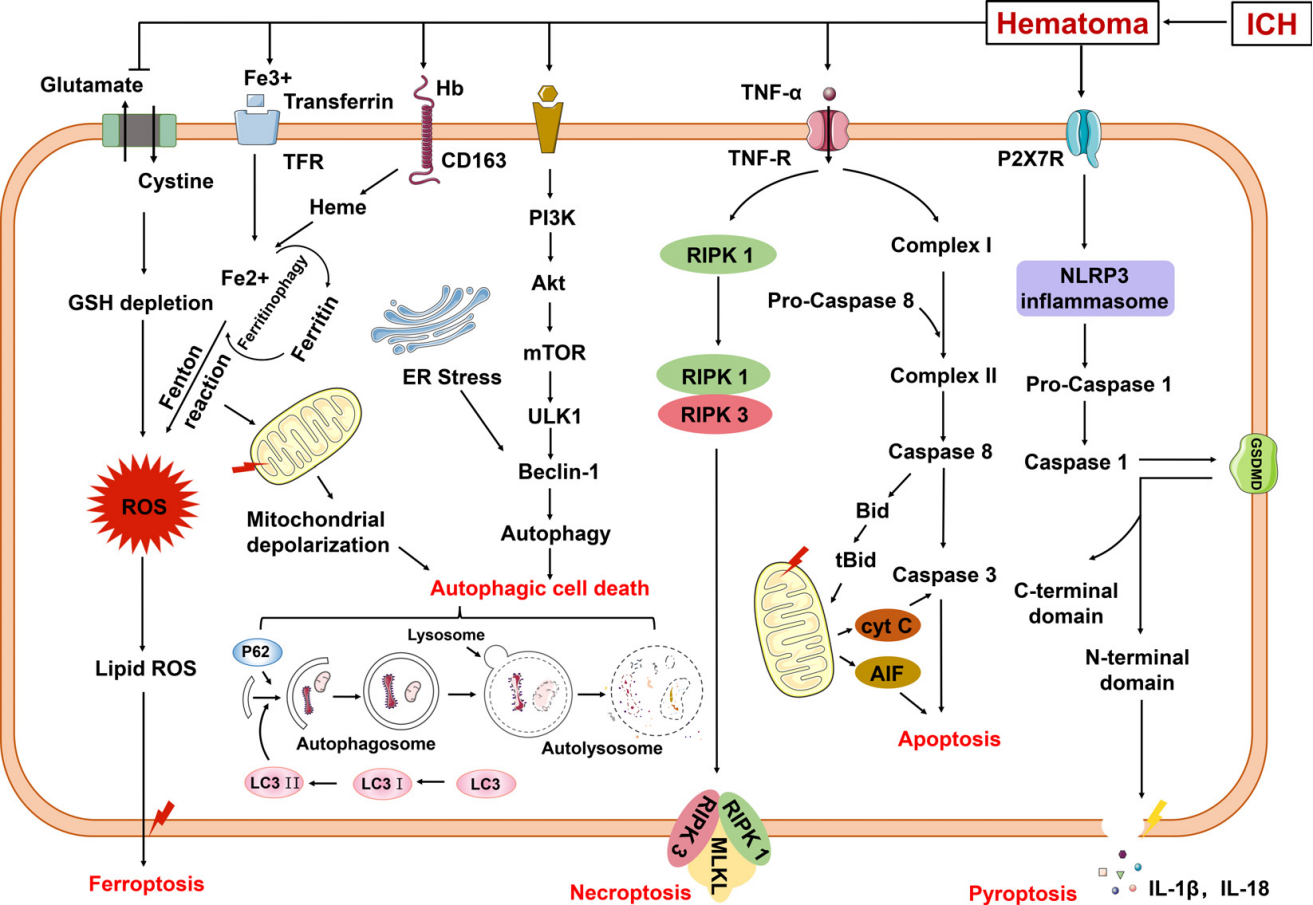
Different regulated cell death mechanisms after ICH. After ICH, blood components are released into brain tissue, which can trigger pro-inflammatory mediators and cause cell death receptor activation. TNF-α triggers RIPK1/FADD/caspase-8-mediated apoptosis or RIPK3/ MLKL-mediated necroptosis. Hematoma formation increases intracranial pressure (ischemia) which increases glutamate levels and thus inhibits system Xc^-^ and triggers ferroptosis. Accumulated iron generates ROS that can cause lipid peroxidation (ferroptosis) and DNA and protein damage (which may also lead to apoptosis or necroptosis). Activated PI3K signaling, ER stress, and mitochondrial depolarization can all trigger autophagy, and excessive autophagy causes autophagic cell death. Blood components can also activate caspase-1 and GSDMD (perhaps through P2X7R), which induces pyroptosis.

Accidental cell death is virtually instantaneous and uncontrollable and occurs under conditions of total ATP depletion and results from external stimuli such as infection, toxins, heat, hypoxia, and trauma [24]. This process is known as necrosis and is generally defined by cytoplasmic swelling, often accompanied by chromatin condensation and an irregular DNA degradation pattern, followed by the breakdown of the plasma membrane and the release of the cell’s contents into the extracellular compartment where they can damage neighboring cells and induce an inflammatory response [25]. In contrast, regulated cell death involves precise signaling cascades, is implemented by a set of defined effector molecules, and has unique biochemical, functional, and immunological consequences. With the rapid progress in the research of regulated cell death, the novel mechanisms that orchestrate multiple cell death pathways have been unveiled and have shown the potential for the treatment of hemorrhagic brain injuries with existing agents. In this review, we summarize the updated concepts of regulated cell death types as well as some potential interventions in preterm infants after ICH (Fig. **2**).

### 3.1 Apoptosis

Apoptosis is a process of programmed cell death mediated by a group of specialized protein-cleaving enzymes called caspases. The morphological features of apoptosis include membrane blebbing, cell shrinkage, nuclear fragmentation, chromatin condensation, chromosomal DNA fragmentation, and the formation of membrane-bound apoptotic bodies [26, 27]. The process of apoptosis includes intrinsic and extrinsic pathways, with the extrinsic pathway being mediated by signaling through membrane-bound death receptors and the intrinsic pathway being mediated by mitochondria-associated stimuli independent of receptor signaling. The ligands and cell membrane death receptors of the extrinsic pathway include Fas ligand/FasR, TNF/TNFR1, Apo2L/DR4, and TNF-related apoptosis-inducing ligand (TRAIL) R1 [27]. All of the intrinsic apoptotic events are primarily controlled by the Bcl-2 family, including pro-apoptotic factors (Bax, Bak, Bid, Bim, Puma, Noxa, Bad, and Blk) and anti-apoptotic factors (Bcl-2, Bcl-XL, Bcl-X, and BAG), which determine the membrane integrity of the mitochondria through the release of intermembrane space proteins such as cytochrome c, Smac/DIABLO, and Omi/Htra2 that trigger or enhance caspase activation or AIF and endonuclease G that acts in a caspase-independent manner [27, 28].

Apoptosis has been verified by detecting apoptotic markers in autopsy tissues and by clinical data from premature infants. Increased soluble Fas (CD95/Apo-1), soluble Fas ligand, and activated caspase-3 have been found in the CSF, and caspase-3 signals have been detected by immunostaining in the periventricular zone of premature infants (25 weeks) with intraventricular hemorrhage, but not in infants without intraventricular hemorrhage (24 weeks) [29, 30]. Apoptosis has also been identified in different neonatal animal models of ICH according to its morphological and molecular characteristics. In glycerol-induced preterm ICH rabbits, apoptosis was indicated by increased cleaved caspase-3 or increased caspase-3/7 activity in the choroid plexus. Furthermore, exposing primary human choroid plexus epithelial cells to CSF derived from rabbit intraventricular hemorrhage models showed small but dose-dependent cell death at 4 hours and almost complete cell death after 24 hours [9]. To further characterize the cellular and molecular mechanisms behind this observation, heme and methemoglobin, the ferric state (Fe^3+^) of hemoglobin, were applied to human choroid plexus epithelial cell culture. Interestingly, methemoglobin could upregulate the activity of caspase-3/7 [9]. In addition, treating cultured primary neurons with oxyhemoglobin increased caspase-3 activation, which suggests that hemoglobin-induced cell death occurs at least partly through apoptosis signaling [9].

### 3.2 Necroptosis

The term “necroptosis” was coined with the identification of the pharmacological receptor-interacting serine/threonine-protein kinase 1 (RIPK1) inhibitor necrostatin-1 (Nec-1) [31]. Although necroptosis and necrosis have similar morphological characteristics (increase in cell volume, organelle swelling, cellular collapse, deficiency of nuclear chromatin, loss of cell membrane integrity, and release of cellular contents), necroptosis is regulated by a series of signal transduction pathways and is a process of positive consumption of energy known as programmed necrosis. Necroptosis is induced by a variety of signals, including death receptor ligands, such as Fas ligand/FasR, TNF/TNFR1, and (TRAIL) R1, which we mentioned above regarding apoptosis. When these death receptor ligands bind, they can activate RIPK1, and in the case of caspase-8 inactivation, activated RIPK1 recruits and phosphorylates RIPK3, which further recruits and phosphorylates MLKL to form the necrosome complex. Phosphorylated MLKL then oligomerizes through its N-terminal four-helix bundle, and this causes its translocation to the plasma membrane and triggers necroptosis [32, 33].

Ultrastructural analyses of injured cells 24 hours after ICH in adult mice showed that the injured striatum had reduced nuclear membrane integrity, swollen mitochondria, and nuclear karyorrhexis, all of which are morphological characteristics of necrosis/necroptosis [34]. Increased expression of RIPK1 and increased interactions of RIPK1, RIPK3, and MLKL were observed in brain tissue after ICH in adult rats suggesting that the formation of the necrosome was significantly increased and necroptosis was activated [35]. Pharmacological inhibition of RIPK1 with Nec-1 or genetic knockout of *Ripk1* decreased RIPK1–RIPK3 interactions, reduced cell death, alleviated inflammation, attenuated edema development, and improved neurobehavioral outcomes after ICH [35, 36]. Knock-out of the necroptosis executor MLKL reduced neuronal death and blood-brain barrier (BBB) permeability and improved post-injury motor function performance [37]. Moreover, an *in vitro* study showed that free hemin mediates neuronal necroptosis in primary cultures of hippocampal neurons by inducing the assembly of the necrosome complex and thus triggering cell death [38]. In addition, inhibiting RIPK1 by Nec-1 or silencing of RIPK3 using siRNA attenuated hemin-induced cell death, thus dramatically improving cell viability and decreasing reactive oxygen species (ROS) accumulation [39]. Another study using the same *in vitro* model also supports this conclusion, and besides the specific inhibitors of apoptosis (z-VAD-fmk) and necroptosis (Nec-1), the autophagy inhibitor 3-Methyladenine (3-MA) was also included to evaluate cell death by the MTT assay after treating HT22 cells with hemin. The results showed that only Nec-1 could reduce cell death, and thus it seems that under these conditions necroptosis was the dominant cell death type rather than apoptosis or autophagy [40]. To explore the mechanism of necroptosis after ICH, oxygen hemoglobin (OxyHb) was used to directly affect neurons or stimulate microglia, and the supernatant was used as the conditioned medium and was collected and used to treat neurons. The results showed that there was a higher apoptotic ratio in the neurons directly stimulated by OxyHb, whereas there was a higher necroptotic ratio in the group treated with a conditioned medium. Furthermore, pretreatment with TNF-α inhibitor reduced the percentage of necroptotic neurons induced by the conditioned medium, which suggests that OxyHb induces microglia to release inflammatory cytokines (TNF-α) into the conditioned medium that in turn initiates necroptosis in neurons [35]. These results indicate that hemin, hemoglobin, and TNF-α can induce neuronal necroptosis. Furthermore, perampanel treatment downregulated the protein expression of RIPK1, RIPK3, and MLKL, as well as IL-1β, IL-6, TNF-α, and NF-κB in an ICH mouse model. These results suggest that glutamate may influence necroptosis after ICH because perampanel is a non-competitive antagonist of the AMPA (α-amino-3-hydroxy-5-methyl-4-isoazolepropionic acid) receptor, which is a subtype of ionotropic glutamate receptor [40]. However, whether glutamate directly induces necroptosis after ICH requires further exploration, and how it influences necroptosis regulator proteins is still unclear, while other studies indicate that glutamate can also induce other forms of cell death such as ferroptosis [41]. These results from studies of *in vitro* cell culture or *in vivo* ICH models suggest that hemin, hemoglobin, TNF-α, and glutamate can induce necroptosis after cerebral hemorrhage, which provides evidence to speculate that necroptosis is one of the cell death forms in preterm infants after ICH.

### 3.3 Pyroptosis

Pyroptosis is a type of regulated cell death that critically depends on the formation of plasma membrane pores by members of the gasdermin protein family, often (but not always) as a consequence of inflammatory caspase activation [42]. Pyroptosis is typically divided into the canonical inflammasome pathway and the noncanonical inflammasome pathway according to whether it depends on caspase-1 [43, 44]. In the canonical inflammasome pathway, the inflammasome is assembled to recognize pathogen-associated molecular patterns and danger-associated molecular patterns by the inflammasome sensors NLRP1, NLRP3, NLRC4, AIM2, and pyrin. NLRP3, AIM, and pyrin activate caspase-1 by recruiting the inflammasome adaptor protein ASC (apoptosis-associated speck-like protein containing a CARD) while NLRP1 and NLRC4 activate caspase-1 directly or by recruiting ASC. Activated caspase-1 promotes the maturation of the precursors of IL-1β and IL-18 and cleaves gasdermin D (GSDMD). In the caspase-1 independent pathway, noncanonical inflammasomes form under-stimulation (such as lipopolysaccharide), then recruit and activate caspase-4 and caspase-5 in human or caspase-11 in the mouse. Both activated caspase-1 and activated caspase-11/4/5 cleave GSDMD into GSDMD-C and GSDMD-N, the latter of which forms pores on the plasma membrane to induce pyroptosis [43, 44]. In addition to the canonical and non-canonical inflammasome pathways, pyroptosis is initiated by other caspases. In some cells with high level of gasdermin E (GSDME), activated caspase-3 can cleave GSDME to generate N-terminal domains, which subsequently form transmembrane pores eventually resulting in pyroptosis [45].

Recent reports have shown that pyroptosis is involved in the pathogenesis of hemorrhagic brain injury. Upregulated NLRP3, cleaved caspase-1, and IL-1β were observed in an adult ICH rodent model, while knocking down NLRP3 inhibited inflammasome formation, reduced caspase-1 and IL-1β production, and led to reduced brain edema and improved neurological functions [46, 47]. Furthermore, P2X7R may induce ONOO (-), a potential downstream signaling molecule of P2X7R and thus might play a critical role in triggering NLRP3 inflammasome activation [47]. In addition to NLRP3, NLRP1 inflammasome-mediated pyroptosis is also involved in ICH, and the melanocortin 4 receptor agonist RO27-3225 successfully attenuated NLRP1-dependent neuron pyroptosis after ICH in mice [48]. Another study on the effect of CCR5 (C-C chemokine receptor 5)-mediated inflammation on cerebral hemorrhage showed that CCR5 and NLRP1 increased after ICH. Inhibition of CCR5 improved the short-term and long-term neurobehavioral deficits and decreased neuronal pyroptosis, and this was accompanied by decreased expression of NLRP1, ASC, caspase-1, GSDMD, and interleukin-1β/IL-18 [49]. Compared with other studies that only focus on inflammasome formation and cytokines release, this study showed the change in GSDMD, the key protein of the canonical inflammasome pathway, and thus provides strong evidence for the occurrence of pyroptosis after cerebral hemorrhage.

There are no reports about pyroptosis in caspase activation and recruitment in neonatal ICH. However, based on the evidence of increased inflammatory cytokines in the immature brain after ICH, including IL-1β, which is an inflammatory cytokine that is cleaved and activated by caspase-1 to form a 31 kDa precursor [14, 50, 51], we speculate that pyroptosis plays a role in the secondary injury in the immature brain after ICH.

### 3.4 Ferroptosis

Ferroptosis is a newly identified form of regulated cell death that is morphologically and biochemically distinct from other types of regulated cell death such as apoptosis, autophagy, and necroptosis [41]. The morphological characteristics of ferroptosis show obvious changes in mitochondria, including a shrinking mitochondrial membrane, increased membrane density, reduced or complete absence of mitochondrial cristae, and outer mitochondrial membrane rupture [41, 52]. Unlike apoptosis or necrosis, cells undergoing ferroptosis still show an intact cell membrane without ruptures or blebs and normal nucleus size with no chromatin condensation or fragmentation [52]. It has been widely accepted that ferroptosis is an iron-dependent and lipid peroxidation-induced cell death pathway that is tightly regulated by a series of genes and various metabolic pathways.

Although the role of iron in ferroptosis remains unclear, iron accumulation generates a large amount of ROS through the Fenton reaction and is a vital inducer of ferroptosis. Cellular iron homeostasis is tightly controlled by a series of iron-binding proteins and by iron movement between different cellular spaces. Under normal physiological conditions, most iron is taken up through the transferrin-transferrin receptor (Tf-TfR) system. Tf binds to Fe^3+^ and undergoes endocytosis after binding to membrane-bound TfR. The endosome releases Fe^3+^ and reduces it to Fe^2+^ *via* divalent metal transporter 1 (DMT1), whereafter Fe^2+^ is stored in ferritin. When ferritin levels become saturated, iron can be transported out into the cerebral interstitial fluid by ferroportin 1 [53]. Under pathological conditions, free iron released from iron-binding proteins causes large amounts of ROS that greatly challenge cellular antioxidant capacity. As the most important cellular antioxidant, the deficiency of glutathione (GSH) will directly promote the generation of lipid peroxidation, which is highly dependent on the selenoenzyme glutathione peroxidase (GPX4). GPX4 converts toxic lipid hydroperoxides into nontoxic lipid hydroxy derivatives by using GSH as a substrate. Besides ROS, lipid peroxidation can also be produced by some dioxygenase homologs, namely lipoxygenases, and there is evidence for their role in ferroptosis. According to lipidomics analysis, polyunsaturated fatty acids are the most susceptible lipids to peroxidation during ferroptosis compared to other classes of lipids. A cluster of proteins linked to the metabolism of polyunsaturated fatty acids such as Acyl-CoA synthetase long-chain family member 4 (ACSL4), 1-acylglycerol-3-phosphate O-acyltransferase 3 (AGPAT3), and lyso-phosphatidylcholine acyltransferase-3 (LPCAT3) have been shown to modulate the susceptibility of cells to ferroptosis [54].

It seems that the brain is vulnerable to ferroptosis because it contains the highest lipid content next to adipose tissue [55]. In fact, ferroptosis is highly related to neurological disorders such as neurodegenerative diseases and hemorrhagic brain injury [56]. Once iron is released from hemoglobin after bleeding, the labile cellular iron pool increases directly and soon overburdens the cellular iron scavenging ability and thus causing increased ROS. There is already much evidence suggesting that ferroptosis is involved in ICH. During hemorrhage, a large amount of iron is released from hemoglobin catalyzed by heme oxygenase 1 (HO-1), and inhibition of HO-1 or deletion of HO-1 reduces brain injury after ICH [57]. High serum levels of iron are independently associated with poor outcomes [58], and iron overload has been shown to induce significant perihematomal edema and increase the water content in the brain [59]. Shrunken mitochondria in the neurons of the perihematomal, hippocampal, and cortical tissue were observed 3 hours, 3 days, 6 days, and 7 days after ICH [59, 60]. Several ferroptosis inhibitors, such as ferrostatin 1 (Fer-1), liproxstatin 1 (Lip-1), N-acetylcysteine (NAC), Trolox, and U012 function as antioxidants and have shown neuroprotection in ICH models [41, 61]. Fer-1 prevents neuronal death and reduces iron deposition induced by hemoglobin in organotypic hippocampal slice culture and improves neurologic function in ICH mice. Mechanistically, Fer-1 reduces lipid ROS production and attenuates the increased expression of *PTGS2* (prostaglandin-endoperoxide synthase 2) and its gene product cyclooxygenase-2, suggesting that cyclooxygenase-2 may be a biomarker of ferroptosis [60]. Inhibiting GPX4 by pharmacological inhibition or genetic knockdown aggravated brain injury in rats after ICH [62]. A recent report found that selenium augments the transcriptional response involving GPX4 *via* coordinated activation of the transcription factors TFAP2c (transcription factor AP-2c) and Sp1 to protect neurons from ferroptosis and to improve functional recovery after ICH in mice [63]. A study has shown that NAC, a clinically approved thiol-containing compound, prevents hemin-induced ferroptosis in primary cortical neuronal cells and improves functional recovery after ICH in mice by neutralizing toxic lipids generated by arachidonate-dependent ALOX5 activity [64], and it functions by increasing GSH levels and neutralizing toxic lipids. In addition, N-hydroxy-N'-(4-n-butyl-2-methyl phenyl)-formamidine (HET0016), which inhibits the synthesis of the arachidonic acid metabolite 20-hydroxyeico-satetraenoic acid (20-HETE), has shown a protective effect after ICH [65]. These results indicate that polyunsaturated fatty acid metabolism, especially arachidonic acid metabolism, participates in ICH-induced secondary brain injury.

Brain iron accumulation, bilateral enlargement of the lateral ventricles, and hippocampal brain tissue loss have been observed in an intraventricular hemorrhage rat model [66, 67] along with upregulated HO-1 and ferritin [68]. Furthermore, infusion of hemoglobin and its degradation products into rats causes ventricular dilation and brain damage, while systemic deferoxamine (DFX) treatment reduces ICH-induced ventricular enlargement and hippocampal brain tissue loss and improves neurological behavior [68]. In addition to posthemorrhagic hydrocephalus, periventricular leukomalacia (PVL) is also a significant sequela of preterm ICH. It has been found that large amounts of lipid oxidation products are found in the CSF of infants with PVL [69]. Moreover, oligodendrocyte death in PVL is related to lipid peroxidation, and the cultivation of oligodendrocytes in a cysteine-free medium showed that exhaustion of GSH caused ferroptosis in cells and that administration of Fer-1 could effectively prevent ferroptosis in oligodendrocytes [70]. Taken together, these results provide further support for a possible association between preterm ICH and ferroptosis.

### 3.5 Autophagy

Unlike the aforementioned regulated cell death types, autophagy is not exactly a form of cell death because it functions as a scavenging system that delivers unnecessary or dysfunctional components to the lysosome. However, recent studies have demonstrated that autophagy has a greater variety of physiological and pathophysiological roles than previously believed [71], and autophagy often accompanies cell death following insults. The formation of the autophagosome is the distinguishing feature under transmission electron microscopy and gives the cell a characteristic vacuolated appearance. The core machinery of autophagy is autophagy-related genes that regulate the whole process of autophagy, and 38 autophagy-related genes have been identified as being involved in macroautophagy in yeast [72]. A number of signal transduction pathways are involved in the regulation of autophagy, and many of these pathways initiate autophagy through mTOR kinase, which is a negative regulator of autophagy [73]. The serine/threonine protein kinase ULK1 (unc-51-like kinase 1), which plays a similar role as the yeast Atg1, acts downstream of the mTOR complex. The downstream phosphorylation target of ULK1 is Beclin1, which acts as an integral scaffold for the PI3K complex, thus promoting autophagic protein localization to autophagic vesicles. The critical regulator LC3 is involved in the formation of autophagosome membranes, and the ratio of the two mutually convertible forms LC3-I and LC3-II can be used to estimate the abundance of autophagosomes [73]. Another important autophagosomal marker is the adapter protein p62 (SQSTM1) which mediates the delivery of ubiquitinated cargo to autophagosomes, which are degraded *via* autophagy along with their cargo in autolysosomes [74].

Accumulating data provide evidence that the autophagy machinery can also be recruited to kill cells under certain conditions by generating a caspase-independent form of regulated cell death called autophagic cell death [75]. In many cases, it is acknowledged that this autophagic cell death is cell death with autophagy rather than cell death by autophagy [76]. The role of autophagy in regulated cell death remains controversial, and the core question is whether autophagic activity in dying cells is the cause of death or is an attempt to prevent death.

Clinical studies in ICH patients who were admitted for hematoma evacuation have demonstrated that autophagy occurs in the area around the hematoma with morphological and positive biochemical evidence [77]. Electron microscopy revealed the presence of autophagosomes and autolysosomes in the brain cells and fewer organelles and mitochondrial cristae, together with more cells that were positive for LC3 (microtubule-associated protein 1A/1B-light chain 3), beclin 1, and cathepsin D in the patient group compared with the control group [77]. Preclinical studies verified and further explored the role of autophagy after ICH. In heme-treated primary neurons, an increased number of autophagosomes was seen by electron microscopy and increased autophagic flux was verified by western blot [78]. Further study showed that neuronal cell death was inhibited by the autophagy-specific inhibitor 3-MA and promoted by the autophagy inducer rapamycin [79]. Also, 3-MA significantly decreased the expression of LC3-II and Beclin-1 and maintained p62 at a high level in an ICH mouse model [79].

The above results showed that iron can induce autophagy, and recent studies have indeed shown that autophagy-related cell death participates in hemorrhagic brain injury through degradation of the iron storage protein ferritin, which is referred to as ferritinophagy [80]. Furthermore, the inhibition of autophagy has been shown to play a protective role by inhibiting apoptotic activation [78, 79]. As to whether autophagy aggravates or alleviates brain injury after hemorrhage remains unclear. One study found that autophagy was enhanced after cerebral hemorrhage and was related to inducing caspase-12 mediated apoptosis and secondary brain injury but was protective at 7 days after ICH [81]. These results suggest that autophagy aggravates brain injury in the acute phase and helps remove damaged nerve cells during the recovery period after hemorrhage [81]. Besides iron and endoplasmic reticulum (ER) stress, thrombin can also induce autophagy after hemorrhage, and hirudin, an inhibitor of thrombin, can reduce the level of autophagy [82]. However, pretreatment with 3-MA inhibits thrombin-induced autophagic vacuole formation and exacerbates thrombin-induced cell death [82]. These results suggest that autophagy is involved in thrombin-induced cell death after cerebral hemorrhage but showed a pro-survival role. However, there are no studies regarding autophagy in the immature brain after ICH.

### 3.6 PANoptosis

PANoptosis is a newly identified form of regulated cell death that consists of apoptosis, pyroptosis, and necroptosis occurring simultaneously under pathophysiological conditions [83]. PANoptosis has been confirmed in infectious diseases and diseases associated with inflammation and immune activation [84]. Recent studies showed that PANoptosis-like cell death occurs in retinal neurons following insults [85] and might play a role in cerebral ischemia [15], and young animals with sepsis-associated encephalopathy revealed PANoptosis in cortical nerve cells [86]. Further analysis showed that the pathways of PANoptosis are interconnected by shared regulatory proteins named the PANoptosome, and many of the components have been implicated in neurological disorders [87]. Multiple forms of regulated cell death have been identified in the brain after hemorrhage [88]; however, there are no experimental studies showing evidence of PANoptosis in cerebral hemorrhagic brain injury. The immature brain possesses programmed cell death machinery, and regulated cell death is more pronounced in the brain after insult. ICH induces microglia activation, immune cell infiltration, and the secretion of cytokines, thus hemorrhage-related inflammation plays an important role in neural cell death and brain injury in the immature brain after hemorrhagic insult. PANoptosis is an inflammation-regulated cell death pathway [84], suggesting that PANoptosis is very likely to be involved in neural cell death in immature brain hemorrhage.

### 3.7 Crosstalk between Different Types of Regulated Cell Death

**Fig. (3):**
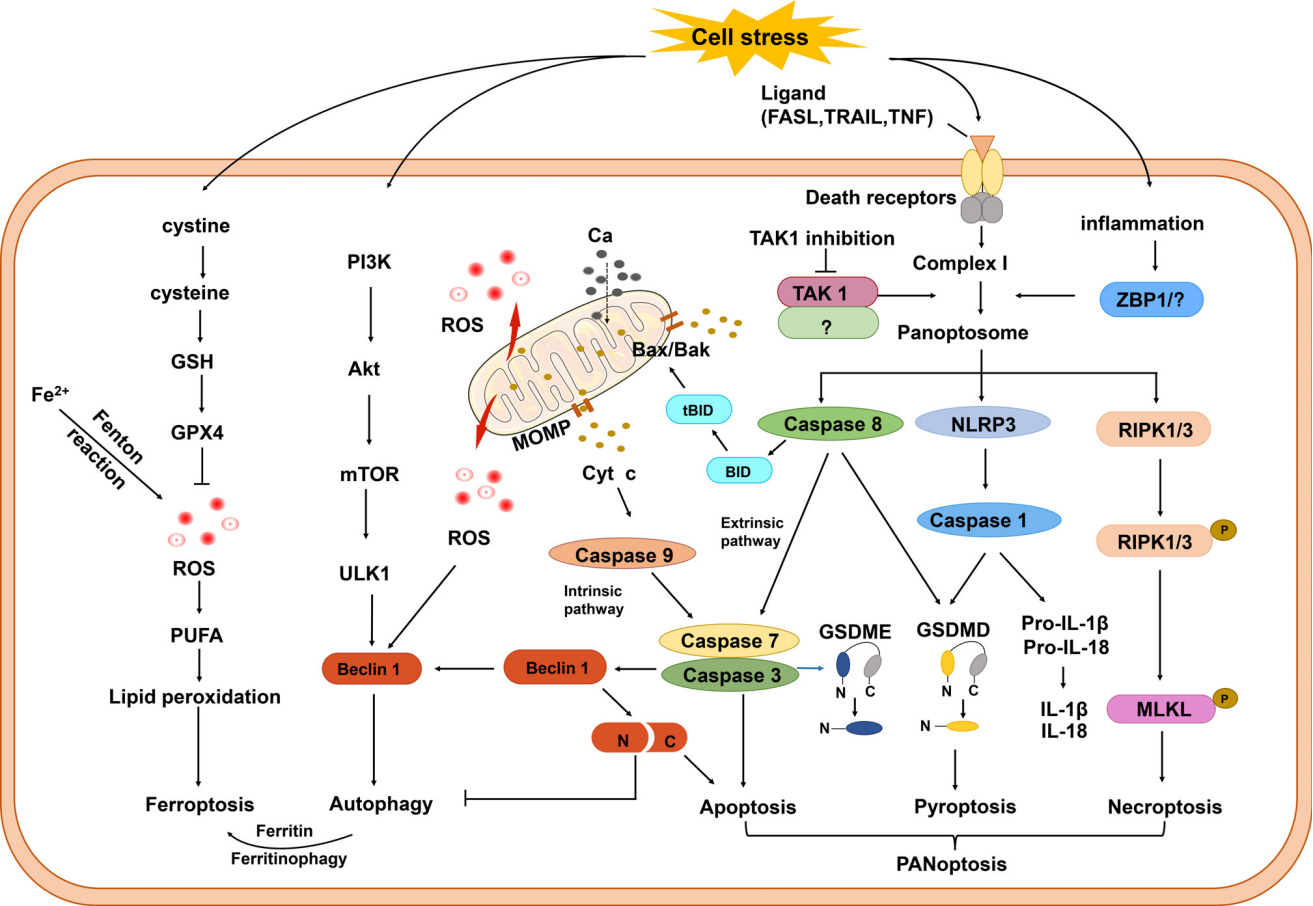
Crosstalk between different cell death pathways. When cells are under overwhelming stress, different programmed cell death pathways will be initiated, and some key regulators play an important role in switching between different programmed cell death pathways. Activation of ZBP1 or inhibition of TAK1 triggers the assembly of the PANoptosome to activate PANoptosis. The main components of the PANoptosome include RIPK1/3, caspase-8, and NLRP3, which are key regulators of necroptosis, apoptosis, and pyroptosis, respectively. Activated RIPK1/3 recruits MLKL and phosphorylates MLKL ultimately leading to necroptosis through plasma membrane disruption and cell lysis. Caspase-8 drives apoptosis by activating caspase-3/7 either through intrinsic or extrinsic pathways. NLRP3 recruits ASC and pro-caspase-1, causing activation and maturation of caspase-1, which then cleaves GSDMD, pro-IL-1β, and pro-IL-18 and thus triggers pyroptosis and releases IL-1β and IL-18. Activated caspase-3 can also induce pyroptosis by cleaving GSDME. Beclin-1 is a switcher protein of autophagy and apoptosis, and full-length Beclin-1 can recruit autophagy-related proteins to form autophagosomes, while cleaved Beclin-1 inhibits autophagy and promotes apoptosis. Free iron released from ferritin by autophagy (termed ferritinophagy) contributes to ROS generation and ultimately induces ferroptosis *via* lipid peroxidation.

Regulated cell death occurs in space and time-limited manner during normal brain development, and abnormal activation of regulated cell death pathways is a common feature of neurological diseases. The molecular mechanisms underlying these distinct forms of cell death are not independent, and recent evidence indicates that there are complex interactions among them (Fig. **3**), and it is suggested that the crosstalk between these processes is the main cause of neuronal death [13]. Further studies on the mechanism of these molecules have provided potentially important discoveries for crosstalk-targeted therapy in neurological diseases [89-91]. The pyroptosis and apoptosis pathways are closely interconnected and are mutually regulated on different levels from pathway initiation to final execution. Both genetic and biochemical data suggest that the caspase-8/RIPK1 platform is critical in the regulation of apoptosis, pyroptosis, and necroptosis [91]. Therefore, caspase-8 represents the molecular switch that controls apoptosis, necroptosis, and pyroptosis and prevents tissue damage [92]. The crosstalk between necroptosis and ferroptosis has been elucidated in multiple sites [93], and necroptosis inhibition can suppress apoptotic and autophagic pathways [90]. These studies suggest the tight interplay between the different molecular mechanisms regulating the signaling pathways of regulated cell death, and the different cell death processes might modulate each other *via* mutual mechanisms. It is therefore of major importance to identify molecular master switches and key decision points of regulated cell death pathways after ICH in the immature brain.

## 4 POTENTIAL INTERVENTIONS TARGETING REGULATED CELL DEATH FOR PRETERM ICH

**Fig. (4):**
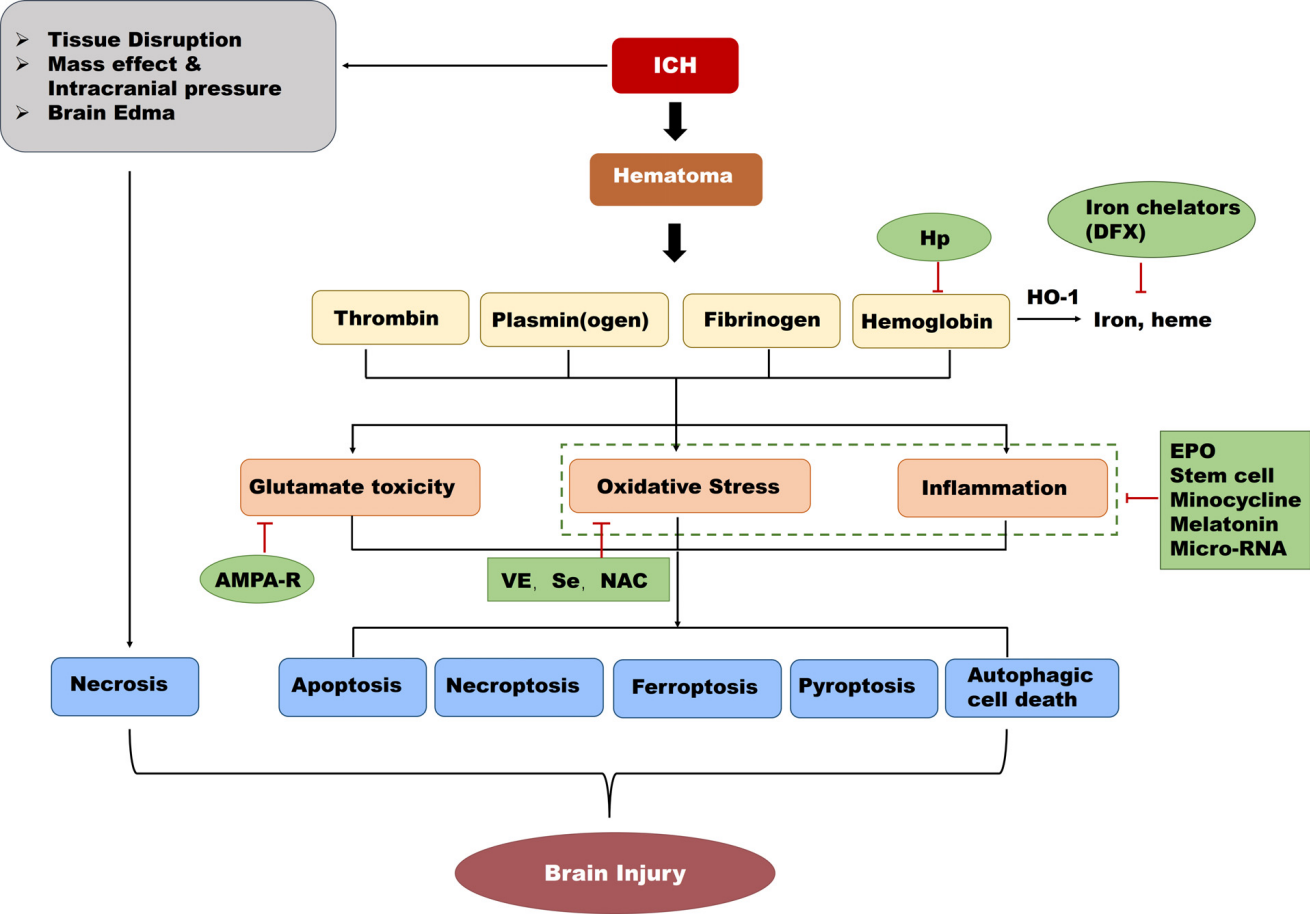
Potential regulated cell death interventions for treating preterm infants with ICH. After ICH, hematoma and blood components cause glutamate toxicity, oxidative stress, and inflammation. Agents in the green ovals show neuroprotective roles by targeting specific processes as shown by red lines. **Abbreviations**: Hp = Haptoglobin, DFX = Deferoxamine, EPO = Erythropoietin, VE = Vitamin E, NAC = N-acetylcysteine.

With improvements in medical and nursing interventions, the incidence of ICH seems to be decreasing in recent decades in developed regions [94]; however, cases of ICH in newborns are inevitable, and there are currently no widely accepted interventions to prevent ICH or treat hemorrhagic brain injury and its neurological sequelae. Here we focus on some potential strategies and their roles in neuroprotection after ICH by influencing regulated cell death (Fig. **4**).

### 4.1 Minocycline

Minocycline is a broad-spectrum tetracycline antibiotic with the ability to cross the BBB. It has been increasingly recognized for its neuroprotective potential and is safe and efficacious for schizophrenia [95]. Minocycline treatment significantly reduced brain iron and brain iron handling proteins (HO-1 and ferritin) after ICH and reduced ICH-induced brain edema, hydrocephalus, and brain damage [96]. Furthermore, as a potent matrix metalloproteinase inhibitor that plays a role in inflammation, minocycline could also protect the neonatal rat brain from ICH injury by inhibiting neuroinflammation [97]. Minocycline was found to inhibit apoptosis and autophagy after ICH [98], which leads to a reduction of brain injury in autologous blood-induced adult rat models. In addition, minocycline demonstrated efficacy and safety in acute stroke patients [99], and thus it is a potential agent for improving the outcomes of premature infants with ICH.

### 4.2 Deferoxamine

DFX is an approved drug for use in other diseases to remove excess iron from the body. It binds to free iron in a stable complex, preventing it from engaging in chemical reactions. Treatment with DFX significantly reduced ferritin levels in the periventricular area at 7 days after ICH in rats, and the effect lasted for 28 days, which reduced white matter loss, basal ganglia loss, post-hemorrhagic ventricular dilation, and cortical loss and improved motor and cognitive function no matter if the treatment with DFX was in the acute or chronic phase [100]. A phase II clinical trial has concluded that DFX-treated subjects have good clinical outcomes at 3 months [101]. However, the forms of cell death that are influenced by DFX after ICH remain enigmatic.

### 4.3 Melatonin

Melatonin is a pleiotropic molecule with diverse physiological functions and in addition to its role in circadian rhythms it also shows anti-oxidant, anti-cell death, and anti-inflammation properties. Various studies have shown that melatonin treatment significantly improves both behavioral and pathological outcomes in animal models of ICH [102]. Melatonin treatment attenuated the expression of IL-1β, IL-18, cleaved caspase-1, and NLRP3 and inhibited inflammasome activation in thrombin-stimulated BV2 cells and improved BV2 cell viability, and incubating HT22 cells with conditioned medium from the BV2 cells also inhibited HT22 apoptosis [103]. It has been shown that melatonin treatment reduces cell apoptosis, inflammation, oxidative stress, DNA damage, brain edema, and BBB damage in the adult ICH model [104]. Furthermore, systemic melatonin treatment ameliorated cognitive and sensorimotor dysfunction at the juvenile developmental stage in a collagenase-induced P7 neonatal rat ICH model, and melatonin also normalized brain atrophy, splenomegaly, and cardiac hypertrophy consequences 1 month after injury [105]. These results suggest that melatonin is an effective antioxidant that can protect the infant brain from the post-hemorrhagic sequelae of mental retardation and cerebral palsy.

### 4.4 AMPA-R Antagonist

AMPA-R, as one of the glutamate receptors, was inhibited in a premature rabbit model of intraventricular hemorrhage as a way to reduce glutamate toxicity and promote neural functional recovery [51]. Perampanel is an FDA-approved antiepileptic that has shown efficacy and safety in phase III clinical trials as a treatment for partial seizures [106]. The mechanism of perampanel-mediated neuroprotection involves anti-cell death, anti-oxidation, and anti-inflammation. As mentioned before, perampanel treatment inhibited necroptosis and inflammatory cytokines, thus alleviating ICH-induced brain injury in mice [40].

### 4.5 Stem Cell Therapy

Stem cell therapy has shown a promising role in preterm infant treatment [107]. Mesenchymal stromal cells (MSCs) are the most commonly used stem cells for preclinical and clinical trials because of their ease of isolation and propagation [108]. Stem cells can self-renew and differentiate into mature neural cells, and stem cells secrete growth factors to enhance endogenous stem/progenitor cell proliferation and to promote axon and dendrite growth, thus improving myelination and functional outcome [109]. MSCs can modulate immune cells including microglia and thus ameliorate inflammation, regulate free radical production, and reduce regulated cell death [110]. A phase I clinical trial showed that allogeneic human umbilical cord blood-derived MSC transplantation into nine premature infants with grade 4 intraventricular hemorrhage was safe and feasible (NCT02673788) [111]. A randomized phase II clinical trial has been initiated to determine the therapeutic efficacy of MSC treatment for intraventricular hemorrhage in premature infants (NCT02890953), but no results have been published even though the trial has finished. Due to limited publication of the clinical study, there is no evidence available to illustrate the benefits or harms of stem cell‐based interventions for the treatment or prevention of intraventricular hemorrhage in preterm infants [112]. Thus, further investigations are needed to evaluate its application value in preterm infants suffering from intraventricular hemorrhage.

### 4.6 Erythropoietin

Erythropoietin (EPO) is a cytokine originally recognized for its role in erythropoiesis. Clinical trials have shown that EPO even at a high dose is safe for both term and preterm infants [113-115]. Our previous study showed that low-dose EPO treatment improved neurological outcomes in newborns with hypoxic-ischemic encephalopathy [114]. Further study revealed that prophylactic low-dose EPO reduced the incidence of ICH and improved neurological outcomes in very preterm infants [116]. A recent study reported that EPO treatment for preterm ICH improved poor outcomes [5]. The neuroprotection of EPO has been thought to be attributed to multiple functions such as anti-oxidant, anti-inflammatory, anti-apoptotic, and neurotrophic effects [117]. Even though EPO has been widely applied in preterm infants to prevent anemia, to treat preterm ICH the optimal dose protocol and course as well as the gestational age of the preterm infants need to be investigated further because EPO treatment has shown gestational age-related differences [116, 118, 119].

### 4.7 Others

In addition to the above neuroprotection strategies for inhibiting cell death after preterm ICH, recent studies have revealed new mechanisms of secondary injury after ICH that will give new insights into other potential strategies.

ROS scavengers like vitamin E, glycine [120], NAC [64], and organoselenium compounds show neuroprotective effects in experimental models of ICH [63, 121]. In the blood-induced ICH rat model, glycine treatment significantly reduced neuronal death and brain edema and improved neurobehavioral outcomes [120]. As a precursor of intracellular GSH, NAC increases the GSH level and targets ALOX5-derived reactive lipid species to inhibit ferroptosis [64]. This also suggests that zileuton, a specific inhibitor of 5-lipoxygenase that is FDA approved for asthma, is a very promising drug for treating ICH by targeting ALOX5 and thus inhibiting lipid ROS. Indeed, a preclinical experiment has shown that treatment with zileuton shows neuroprotective effects in mice after ICH [122]. Selenium is indispensable for selenoproteins, specifically GPX4 [63], and a recent study showed that pharmacological administration of selenium increased the transcription of *GPX4* and other genes and effectively inhibited GPX4-dependent ferroptosis as well as cell death induced by excitotoxicity or ER stress [123], thus improving neurological function after ICH [63]. It has been shown that microRNAs have the potential to inhibit or regulate apoptosis, necroptosis, and autophagy, which may provide neuroprotection in ICH. Overexpression of miR-23b [124], miR-152 [125], and miR-26a-5p [125] effectively alleviates neurological deficits, brain edema, and hematoma areas in ICH rats. However, miRNA therapeutics have not yet been translated into FDA-approved candidates for medical interventions. Also, considering the difficulty of delivering drugs into the brain, the possibilities for using microRNA therapy in ICH require further experimental support. Most other drugs, especially specific inhibitors of key regulator proteins of cell death, have shown neuroprotective effects in ICH in preclinical studies, and we expect new findings regarding the application of these agents in the treatment of preterm ICH, which needs further exploration and clinic trials.

## CONCLUSION AND FUTURE PERSPECTIVES

Preclinical and clinical experiments have demonstrated that regulated cell death in preterm brain injuries after ICH involves apoptosis. Whether other cell death signaling pathways are activated after ICH in preterm infants lacks sufficient evidence. This is partly because most studies on ICH failed to identify the types of cells subjected to cell death, while other studies on cell death mechanisms mostly used solely apoptotic markers as indicators of cell death. One reason why past research has mostly focused on apoptosis is that it was the earliest and most widely studied cell death type, and limitations of detection methods and the non-specificity of detection markers have impeded researchers in more clearly distinguishing different types of regulated cell death. With progress in the field of cell death research, more and more studies have observed other kinds of regulated cell death after ICH. Converging the observations from animal models to human brain autopsies, we have reviewed the mechanisms and the potential roles of necroptosis, pyroptosis, ferroptosis, autophagy, and PANoptosis in preterm ICH. In general, the secondary brain injury after ICH is generated mainly by hemoglobin and its degradation products, and various studies have been performed trying to scavenge these toxins and have shown certain neuroprotective effects. We have also summarized the main potential therapeutic strategies and their functions in regulating cell death. Some agents, such as selenium, have shown great value in targeting non-apoptotic cell death. These studies suggest avenues for further exploration of such therapeutic interventions in preterm infants after ICH.

## LIST OF ABBREVIATIONS

AMPA: α-Amino-3-hydroxy-5-methyl-4-isoazolepro-pionic Acid
ASC: Apoptosis-Associated Speck-Like Protein Containing a CARD
BBB: Blood-Brain Barrier
CSF: Cerebral Spinal Fluid
EPO: Erythropoietin
Fer-1: Ferrostatin 1
GPX4: Glutathione Peroxidase 4
ICH: Intracerebral Hemorrhage
LC3: Microtubule-Associated Protein 1A/1B-light chain 3
Lip-1: Liproxstatin 1
3-MA: 3-Methyladenine
MLKL: Mixed Lineage Kinase Domain-like
MSCs: Mesenchymal Stromal Cells
NAC: N-acetylcysteine
Nec-1: Necrostatin-1
OxyHb: Oxygen Hemoglobin
PVL: Periventricular Leukomalacia
RIPK1: Receptor-interacting Serine/threonine-protein Kinase 1
RIPK3: Receptor-interacting Serine/threonine-protein Kinase 3
TNFR1: Tumor Necrosis Factor Receptor 1
TRAIL: Tumor-necrosis Factor-related Apoptosis-inducing Ligand
